# Supplementary material for: Combined Therapy with a CCR2/CCR5 Antagonist and FGF21 Analogue Synergizes in Ameliorating Steatohepatitis and Fibrosis
Source: Int J Mol Sci. 2022 Jun 15;23(12):6696. doi: 10.3390/ijms23126696 (PMC9224277; doi:10.3390/ijms23126696)
Supplement: Supplementary file 1 [file ijms-23-06696-s001.zip › ijms-1730649-supplementary/Figure S1_new.pdf]

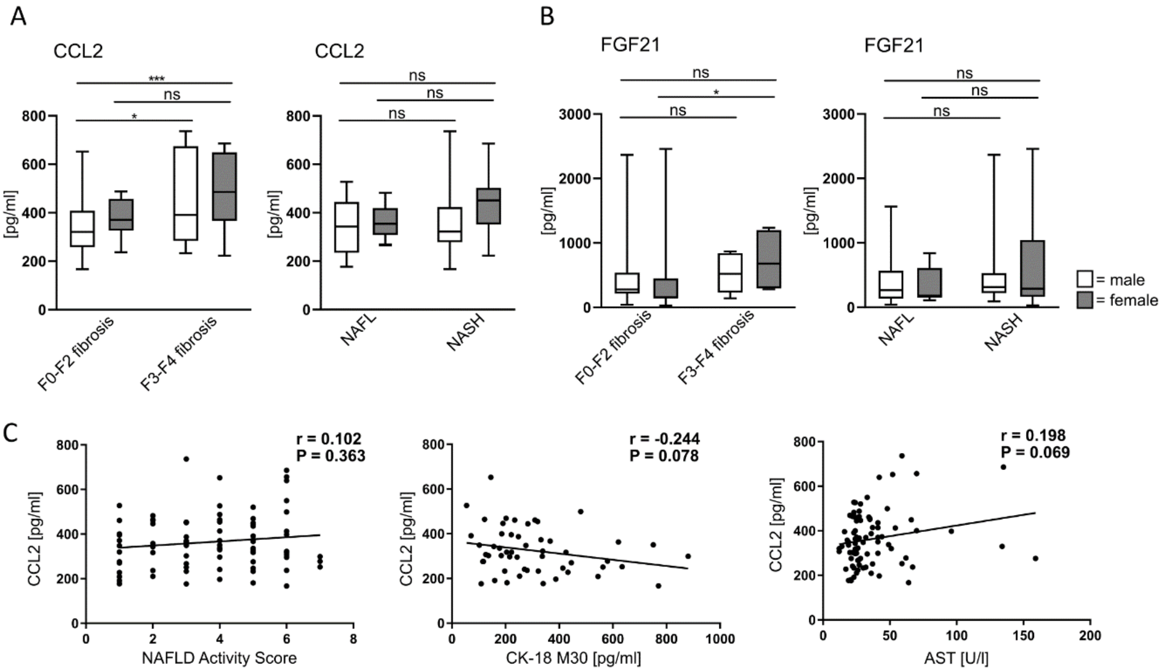


**Supplementary Figure S1.** Gender specific correlation of CCL2 and FGF21 serum levels with severity of human NAFLD. Serum samples were obtained from patients with biopsy-proven NAFLD (n=85). (**A-B**) CCL2 and FGF-21 serum levels measured by ELISA and divided by gender in correlation with histologically assessed severity of liver fibrosis and steatohepatitis. (**C**) Correlation of CCL2 serum concentrations with NAFLD Activity Score (NAS) and biomarkers of fibrosis and liver injury (CK-18 fragment M30 and AST). *P<0.05, **P<0.01, ***P<0.001 (unpaired Student t test in A-B, Spearman's r and P-values of linear correlation analysis in C).
