# Supplementary material for: Combined Therapy with a CCR2/CCR5 Antagonist and FGF21 Analogue Synergizes in Ameliorating Steatohepatitis and Fibrosis
Source: Int J Mol Sci. 2022 Jun 15;23(12):6696. doi: 10.3390/ijms23126696 (PMC9224277; doi:10.3390/ijms23126696)
Supplement: Supplementary file 1 [file ijms-23-06696-s001.zip › ijms-1730649-supplementary/Figure S2_new.pdf]

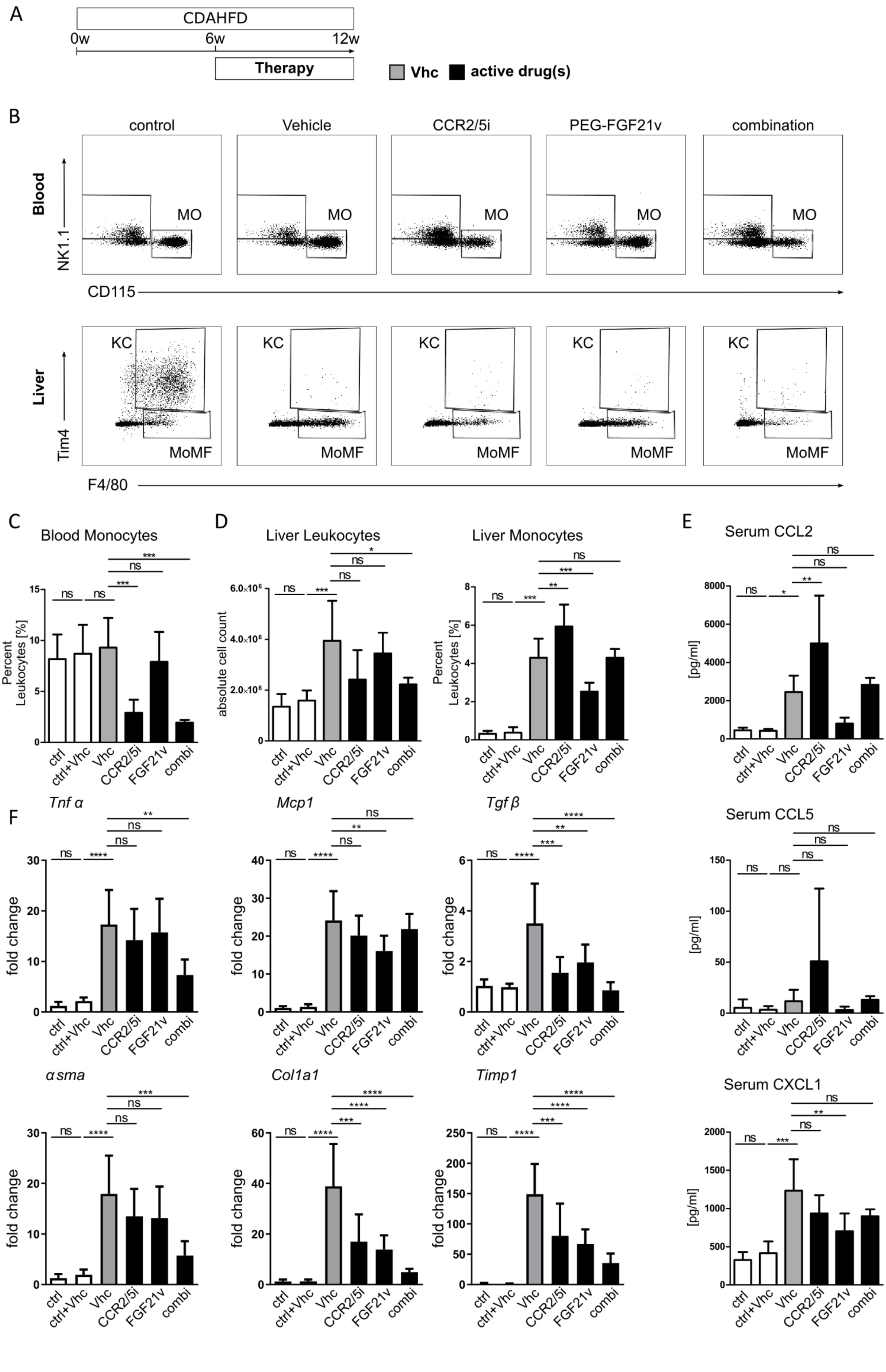
A
CDAHFD
0w
6w
12w
Therapy
Vhc
active drug(s)
B
control
Vehicle
CCR2/5i
PEG-FGF21v
combination
Blood
NK1.1
MO
CD115
Liver
Tim4
KC
MoMF
F4/80
C
Blood Monocytes
Percent Leukocytes [%]
D
Liver Leukocytes
absolute cell count
Liver Monocytes
Percent Leukocytes [%]
E
Serum CCL2
[pg/ml]
Serum CCL5
[pg/ml]
Serum CXCL1
[pg/ml]
F
Tnf α
Mcp1
Tgf β
α sma
Col1a1
Timp1
fold change
ctrl
ctrl+Vhc
Vhc
CCR2/5i
FGF21v
combi
ns
*
**
***
****

Supplementary Figure S2. Effects of combination treatment after long term treatment. (**A**) Chronic liver injury was induced over a total period of 12 weeks and effects of pharmacologic treatment with vehicle (Vhc), CCR2/CCR5 inhibitor (CCR2/5i) and/or PEG-FGF21 variant (FGF21v) was assessed after administration over the last 6 weeks of 12 weeks injury induction. (**B-D**) Representative FACS plots and corresponding quantification of blood (MO = monocytes) and liver immune cells (MoMF = monocyte-derived macrophages, KC = Kupffer cells and leukocytes). (**E**) Quantification of serum CCL2, CCL5 and CXCL1 levels by ELISA. (**F**) Fold change of gene expression levels from total liver tissue measured by RT-qPCR. All data are presented as mean SD (n = 6-8) *P<0.05, **P<0.01, ***P<0.001, ****P<0.0001 (one-way ANOVA with post-hoc testing).
