# Supplementary material for: Combined Therapy with a CCR2/CCR5 Antagonist and FGF21 Analogue Synergizes in Ameliorating Steatohepatitis and Fibrosis
Source: Int J Mol Sci. 2022 Jun 15;23(12):6696. doi: 10.3390/ijms23126696 (PMC9224277; doi:10.3390/ijms23126696)
Supplement: Supplementary file 1 [file ijms-23-06696-s001.zip › ijms-1730649-supplementary/Figure S3_new.pdf]

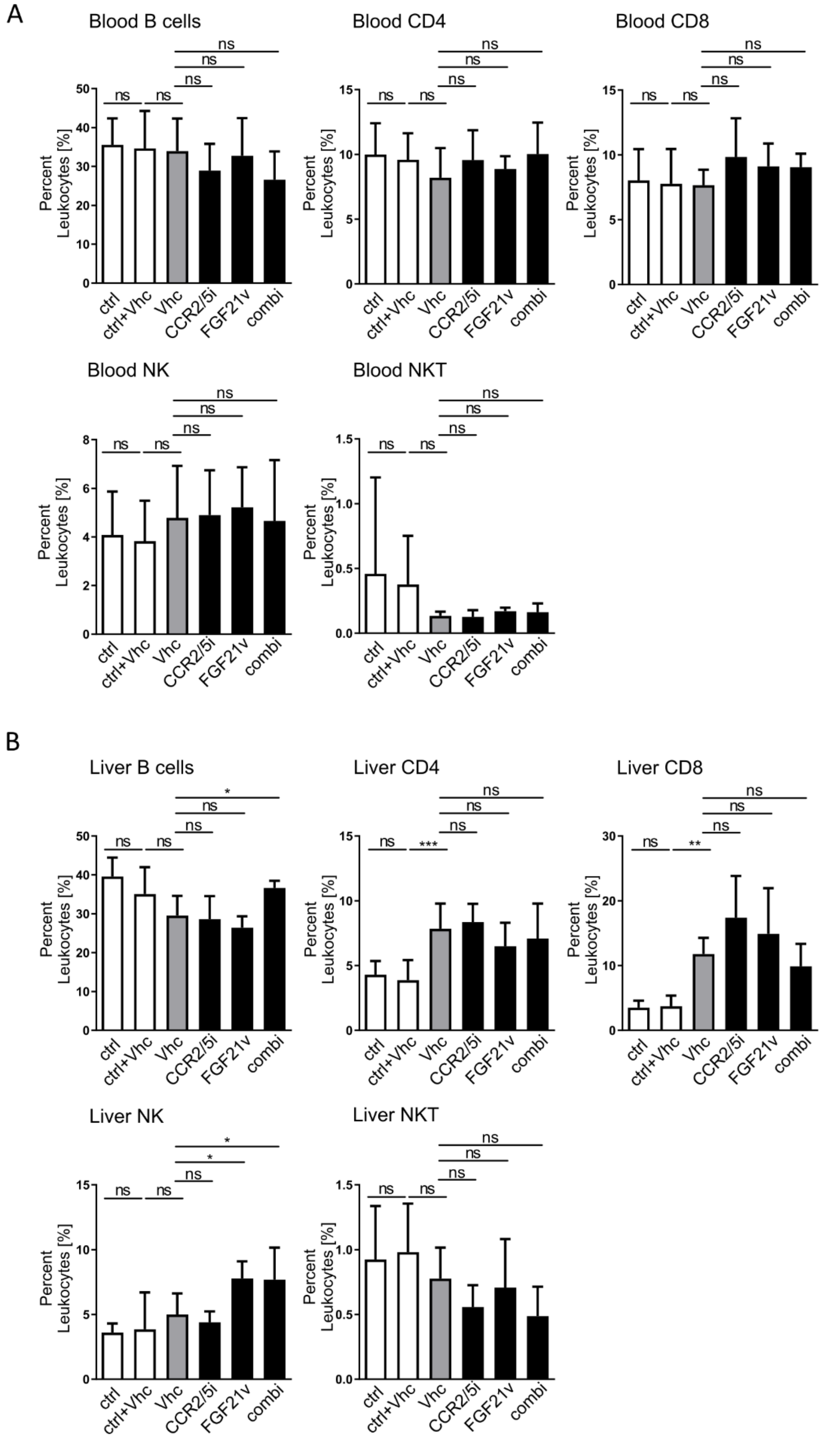

Supplementary Figure S3. Lymphocyte populations in long term chronic liver injury (**A-B**) Quantification of lymphocyte immune cell populations in blood (B cells, CD4+ T cells, CD8+ T cells, NK cells and NKT cells) and liver (B cells, CD4+ T cells, CD8+ T cells, NK cells and NKT cells). All data are

presented as mean SD (n = 6-8) *P<0.05, **P<0.01, ***P<0.001, ****P<0.0001 (one-way ANOVA with post-hoc testing).
