# Supplementary material for: Combined Therapy with a CCR2/CCR5 Antagonist and FGF21 Analogue Synergizes in Ameliorating Steatohepatitis and Fibrosis
Source: Int J Mol Sci. 2022 Jun 15;23(12):6696. doi: 10.3390/ijms23126696 (PMC9224277; doi:10.3390/ijms23126696)
Supplement: Supplementary file 1 [file ijms-23-06696-s001.zip › ijms-1730649-supplementary/Table S1.pdf]

Supplementary Table S1. Histological features according to diagnosis

Histological feature		NAFL (n = 31)	NASH (n = 54)
Steatosis, n (%)	No (< 5%)	0 (0)	0 (0)
	Mild (5-33%)	20 (64.5)	12 (23.1)
	Moderate (33-66%)	7 (22.6)	23 (44.2)
	Severe (≥ 66%)	4 (12.9)	17 (32.7)
Lobular inflammation, n (%)	No foci	24 (77.4)	0 (0)
(per 200x field)	< 2 foci	7 (22.6)	38 (73.1)
	2-4 foci	0 (0)	14 (26.9)
	> 4 foci	0 (0)	0 (0)
Ballooning, n (%)	None	29 (93.6)	0 (0)
	Few ballooning cells	1 (3.2)	30 (57.7)
	Prominent ballooning	1 (3.2)	22 (42.3)
Fibrosis, n (%)	Stage 0 (none)	14 (45.2)	2 (3.7)
	Stage 1 (perisinusoidal or periportal)	15 (48.4)	20 (37.0)
	Stage 2 (perisinusoidal and periportal)	2 (6.5)	20 (37.0)
	Stage 3 (bridging fibrosis)	0 (0)	9 (16.7)
	Stage 4 (cirrhosis)	0 (0)	3 (5.6)

* Data are shown in absolute numbers and % (in parenthesis).
NAFL: non-alcoholic fatty liver; NASH: non-alcoholic steatohepatitis
