# Supplementary material for: Combined Therapy with a CCR2/CCR5 Antagonist and FGF21 Analogue Synergizes in Ameliorating Steatohepatitis and Fibrosis
Source: Int J Mol Sci. 2022 Jun 15;23(12):6696. doi: 10.3390/ijms23126696 (PMC9224277; doi:10.3390/ijms23126696)
Supplement: Supplementary file 1 [file ijms-23-06696-s001.zip › ijms-1730649-supplementary/Table S2.pdf]

Supplementary Table S2. Univariate analysis according to the presence of advanced (≥F3) Fibrosis

	**≤F2 Fibrosis (n=73)**	**≥F3 Fibrosis (n=12)**	**P value**
Age, years	44,8 ± 11,7	55,8 ± 9,6	0.003**
Type 2 diabetes, %	26 (36)	9 (75)	0.011*
ALT, U/l	37 (31,5-62)	44 (32-70)	0.883
AST, U/l	26 (23-41)	36 (27-58)	0.130
AST/ALT ratio	0.73 ± 0.2	0.92 ± 0.27	0.005**
GGT, U/l	33 (23-56)	92 (48-145)	0.006**
Thrombocytes, x $10^3/\mu l$	225 (198-270)	200 (171-277)	0.436
Total cholesterol, mg/dl	174 (150-211)	153 (98-235)	0.351
Triglycerides (mg/dl)	174 (129-218)	230 (170-452)	0.264
CCL2, pg/ml	345,6 ± 102,4	469,7 ± 117,7	<0.001***
FGF-21, pg/ml	260 (157-537)	674 (281-868)	0.07

* Results are expressed as mean ± SD or median (interquartile range) for continuous variables, depending on the normality of the distribution, and n (%) for categorical variables. * P < 0.05; ** P < 0.01; *** P < 0.001.
ALT: Alanine Aminotransferase; AST: Aspartate Aminotransferase; CCL2: chemokine (C-C motif) ligand 2; FGF-2: fibroblast growth factor 21; GGT: γ-glutamyltransferase.
