# Supplementary material for: Combined Therapy with a CCR2/CCR5 Antagonist and FGF21 Analogue Synergizes in Ameliorating Steatohepatitis and Fibrosis
Source: Int J Mol Sci. 2022 Jun 15;23(12):6696. doi: 10.3390/ijms23126696 (PMC9224277; doi:10.3390/ijms23126696)
Supplement: Supplementary file 1 [file ijms-23-06696-s001.zip › ijms-1730649-supplementary/Table S3.pdf]

Supplementary Table S3. Multivariate analysis according to the presence of advanced (≥F3) Fibrosis

	≥F3 fibrosis (n=12) vs. ≤F2 fibrosis (n=73)		
	**OR**	**95% CI**	**P value**
Ratio AST/ALT	15.981	1.052-242.861	0.046*
Type 2 diabetes, presence	5.131	1.096-24.020	0.038*
CCL2, pg/ml	1.007	1.001-1.013	0.017*

* $P < 0.05$. ALT: alanine aminotransferase; AST: aspartate aminotransferase; CCL2: chemokine (C-C motif) ligand 2.
