# Supplementary material for: Combined Therapy with a CCR2/CCR5 Antagonist and FGF21 Analogue Synergizes in Ameliorating Steatohepatitis and Fibrosis
Source: Int J Mol Sci. 2022 Jun 15;23(12):6696. doi: 10.3390/ijms23126696 (PMC9224277; doi:10.3390/ijms23126696)
Supplement: Supplementary file 1 [file ijms-23-06696-s001.zip › ijms-1730649-supplementary/Table S4.pdf]

Supplementary Table S4. Genes and primers used for real-time qPCR on mouse liver tissue

NCBI Gene ID	Gene	Abbreviation	Forward primer	Reverse primer	Role
14433	Glyceraldehyde-3-phosphate dehydrogenase	GAPDH	CATGGCCTTCCGTGTTCCTA	GCGGCACGTCAGATCCA	Housekeeping gene
15452	Hypoxanthine-guanine phosphoribosyltransferase	HPRT	GTTAAGCAGTACAGCCCCAAA	AGGGCATATCCAACAACAAACTT	Housekeeping gene
66945	Succinate dehydrogenase complex, subunit A	SDHA	CTTGAATGAGGCTGACTGTG	ATCACATAAGCTGGTCCTGT	Housekeeping gene
15288	Hydroxymethylbilane synthase	HMBS	AAGGGCTTTTCTGAGGCACC	AGTTGCCCATCTTTCATCACTG	Housekeeping gene
20296	Macrophage Chemoattractant protein-1	MCP-1	TTAAAAACCTGGATCGGAACCAA	GCATTAGCTTCAGATTTACGGGT	Chemokine
21926	Tumor necrosis factor alpha	TNF-α	CATCTTCTCAAAATTCGAGTGACAA	TGGGAGTAGACAAGGTACAACCC	Cytokine
11475	Alpha smooth muscle actin	Acta2	CCAGCACCATGAAGATCAAG	TGGAAGGTAGACAGCGAAGC	Pro-fibrotic gene
21803	Transforming growth factor beta	TGF-β	TGAGCGTCACTGGAGTTGTACGG	GGTTCATGTCATGGATGGTGC	Pro-fibrotic gene
21857	Tissue inhibitor of metalloproteinase 1	Timp 1	CTTGGTTCCCTGGCGTACTC	ACCTGATCCGTCCACAAACAG	Pro-fibrotic gene
12842	Collagen type1 alfa 1	Col1A1	GCTCCTCTTAGGGGCCACT	CCACGTCTCACCATTGGGG	Pro-fibrotic gene
