# Supplementary material for: Combined Therapy with a CCR2/CCR5 Antagonist and FGF21 Analogue Synergizes in Ameliorating Steatohepatitis and Fibrosis
Source: Int J Mol Sci. 2022 Jun 15;23(12):6696. doi: 10.3390/ijms23126696 (PMC9224277; doi:10.3390/ijms23126696)
Supplement: Supplementary file 1 [file ijms-23-06696-s001.zip › ijms-1730649-supplementary/Table S5.pdf]

Supplementary Table S5. Immune cell characterization by flow cytometry.

Immune cells	Surface marker expression profile
Leukocytes	$CD45^{+}$
Granulocytes	$CD45^{+}$, $Ly6G^{high}$, $Gr1^{+}$, $CD31^{-}$, $CD4^{-}$, $CD3^{-}$, $CD19^{-}$
Monocytes	$CD45^{+}$, $Ly6G^{-}$, $CD11b^{high}$, $F4/80^{-}$, $Gr1^{+/-}$, $CD31^{-}$, $CD4^{-}$, $CD3^{-}$, $CD19^{-}$
Monocyte-derived macrophages (MoMF)	$CD45^{+}$, $Ly6G^{-}$, $CD11b^{high}$, $F4/80^{+}$, $Gr1^{+/-}$, $CD31^{-}$, $CD4^{-}$, $CD3^{-}$, $CD19^{-}$
Kupffer cells	$CD45^{+}$, $Ly6G^{-}$, $CD11b^{intermediate}$, $F4/80^{high}$, $Tim4^{+}$, $CD31^{-}$, $CD4^{-}$, $CD3^{-}$, $CD19^{-}$
B cells	$CD45^{+}$, $Ly6G^{-}$, $CD31^{-}$, $TCRb^{-}$, $CD19^{+}$
T cells	$CD45^{+}$, $Ly6G^{-}$, $CD31^{-}$, $CD19^{-}$, $TCR\beta^{+}$
CD4 T cells	$CD45^{+}$, $Ly6G^{-}$, $CD31^{-}$, $CD19^{-}$, $TCR\beta^{+}$, $CD4^{+}$
CD8 T cells	$CD45^{+}$, $Ly6G^{-}$, $CD31^{-}$, $CD19^{-}$, $TCR\beta^{+}$, $CD8^{+}$
NK cells	$CD45^{+}$, $Ly6G^{-}$, $CD31^{-}$, $NK1.1^{+}$, $TCR\beta^{-}$
NKT cells	$CD45^{+}$, $Ly6G^{-}$, $CD31^{-}$, $NK1.1^{+}$, $TCR\beta^{+}$
